# Bilateral Adrenal Leiomyomas in a Pediatric Patient

**DOI:** 10.1210/jcemcr/luaf029

**Published:** 2025-03-07

**Authors:** Ricardo A Caravantes, Miranda Matzer, Mischelle Lopez, Kevin D Santizo, Brígida Hernandez, Isabella Santamarina

**Affiliations:** Department of Medical Research, Universidad Francisco Marroquín, Guatemala City 01010, Guatemala; Department of Medical Research, Universidad Francisco Marroquín, Guatemala City 01010, Guatemala; Department of Medical Research, Universidad Francisco Marroquín, Guatemala City 01010, Guatemala; Department of Research, Universidad de San Carlos de Guatemala, Guatemala City 01010, Guatemala; Department of Research, Universidad de San Carlos de Guatemala, Guatemala City 01010, Guatemala; Department of Medical Research, Universidad Francisco Marroquín, Guatemala City 01010, Guatemala

**Keywords:** adrenal leiomyoma, bilateral adrenalectomy, pediatric adrenal tumor

## Abstract

Adrenal leiomyomas are rare, benign tumors originating in the adrenal glands. They have a varied age of presentation, occur with a slight female predominance, and are typically unilateral, although bilateral cases can occur. Symptoms typically include abdominal or flank pain. This report presents a rare case of an 11-year-old male with disseminated molluscum contagiosum, diagnosed with bilateral adrenal leiomyomas. Imaging revealed large, heterogeneous adrenal masses, and the patient underwent successful adrenalectomy. This case underscores the rarity of adrenal leiomyomas in the pediatric population and highlights the critical role of imaging and surgical intervention in their management.

## Introduction

Adrenal leiomyomas are exceptionally rare benign tumors that originate from the smooth muscle cells of the adrenal glands. With only 28 cases reported in the English literature, these tumors present a unique challenge in clinical practice. They can be diagnosed at a wide range of ages, with a median age of diagnosis of 34.5 years. A slight predominance of female patients has been noted, though both genders can be affected. While these tumors are most often unilateral, bilateral cases have also been documented. Tumor size differs significantly, typically ranging from 3 to 21 cm, with a median of 8 cm [1].

The clinical presentation of adrenal leiomyomas can vary, with abdominal or flank pain occurring in nearly 50% of cases. Other nonspecific symptoms, such as weight loss, anorexia, and malaise, may also be present. Although most adrenal leiomyomas are nonfunctional, some cases involve hormone secretion, with catecholamine production leading to hypertension and elevated levels of norepinephrine and epinephrine [2]. This case highlights the rarity and clinical significance of adrenal leiomyomas in the pediatric population, where they are even less commonly seen. The patient, an 11-year-old male with disseminated molluscum contagiosum, was found to have bilateral adrenal masses. Surgical management through laparoscopic adrenalectomy was initially planned, but due to complications during surgery, an open technique was performed instead.

## Case Presentation

An 11-year-old male with a 1-year history of papular-vesicular lesions was referred by the dermatology department. His mother reported slow-growing papules originating on the lower limbs and spreading across the body, diagnosed as disseminated molluscum contagiosum. Despite cryosurgery, no improvement was observed, prompting referral to a pediatric hospital.

The patient was born prematurely at 7 months of gestation, with a low birth weight of 3 pounds (1.36 kilograms). His neonatal course was complicated by pneumonia, requiring orotracheal intubation for 25 days. During childhood, he experienced 2 episodes of community-acquired pneumonia. His immunizations are up to date.

## Diagnostic Assessment

**Figure 1.**
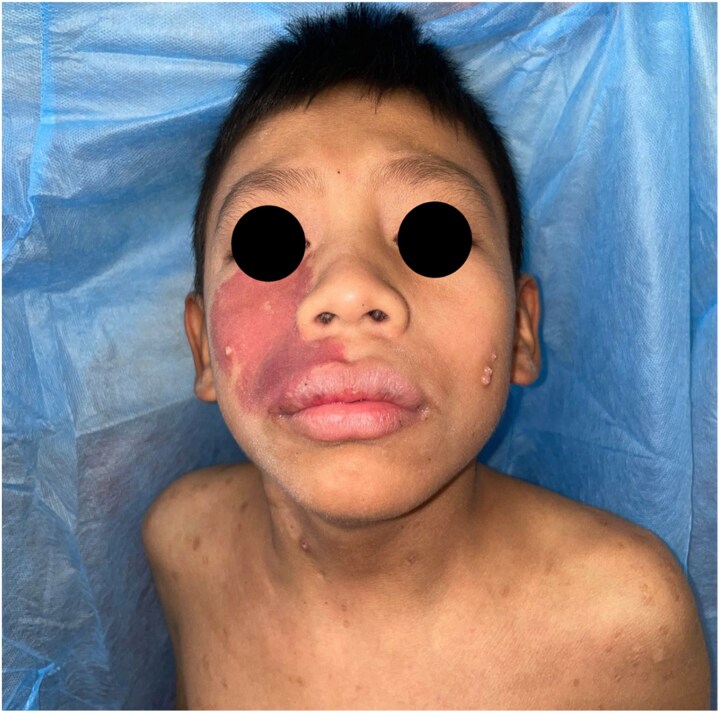
Port-wine stain over the maxillary region and disseminated molluscum lesions.

On physical examination, the patient presented with disseminated dermatosis affecting the head, thorax, abdomen, and both upper and lower extremities. A port-wine stain was noted in the V2 region of the trigeminal nerve [Fig. 1]. Palpation of the abdomen revealed tenderness during both superficial and deep examination.

Initial laboratory tests revealed elevated Epstein-Barr virus (EBV) VCA IgG (63.49 U/mL; reactive >1 U/mL), cytomegalovirus (CMV) IgG (>250 arbitrary unit per milliliter (AU/mL); positive >6 AU/mL), and rubella IgG (134.70 UI/mL; positive >10 UI/mL). Tests for human immunodeficiency virus (HIV) were negative. Immunoglobulin levels (IgM, IgA, IgG) were within normal limits, while IgE was elevated at 1856 UI/mL (4454.4 µg/L), with normal concentrations being <60 UI/mL. Complement levels (C3 and C4) were normal.

**Figure 2.**
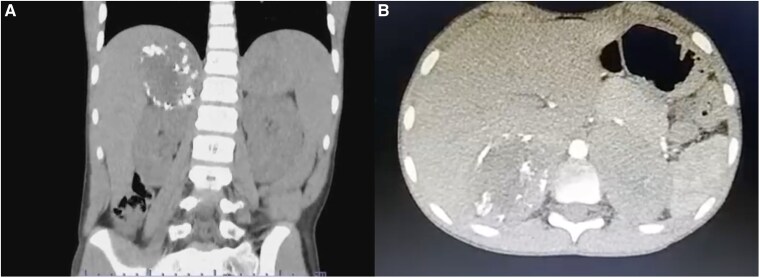
Coronal (A) and axial (B) CT images showing bilateral adrenal masses, with calcification at the periphery of the right adrenal mass.

Due to abdominal pain upon superficial palpation, abdominal ultrasonography was ordered and revealed multiple hepatic lesions, prompting a triphasic liver computed tomography (CT) scan. This imaging ruled out hepatic involvement and revealed bilateral suprarenal masses. Further abdominal CT confirmed a right adrenal mass measuring 5.3 × 5.3 × 4.8 cm with a volume of 70.50 cc and peripheral calcifications, and a left adrenal mass measuring 8 × 6.5 × 6.1 cm with a volume of 165.90 cc with no calcifications [Fig. 2].

Given the imaging and clinical findings, further evaluation was conducted to rule out conditions such as Sturge-Weber syndrome. The hormonal workup—including measurements of vanillylmandelic acid (VMA), catecholamines, metanephrines, adrenocorticotropic hormone (ACTH), and both morning and evening cortisol levels—returned normal results, ruling out functional adrenal tumors [Table 1]. Urinary cortisol levels were not obtained due to their unavailability at the hospital. Cerebral magnetic resonance imaging revealed no abnormalities. Based on these findings, a diagnosis of bilateral adrenal malignancy was considered.

**Table 1.** Hormonal workup and normal reference ranges

Hormonal workup	Patient values (conventional | international system of units)	Normal range (conventional | international system of units)
Vanillylmandelic acid	5.63 mg/24 hours | 30.90 µmol/24 hours	1.90-9.80 mg/24 hours | 10.40-53.80 µmol/24 hours
Catecholamines	51 mg/24 hours | 0.28 µmol/24 hours	< 100 mg/24 hours | < 0.55 µmol/24 hours
Metanephrines	0.41 mg/24 hours | 2.25 µmol/24 hours	< 1 mg/24 hours | < 5.50 µmol/24 hours
Adrenocorticotropic hormone	41.89 pg/mL | 0.042 ng/mL	7.20-63.30 pg/mL | 0.007-0.063 ng/mL
Morning cortisol	15.70 µg/dL | 432.30 nmol/L	3.70-19.40 µg/dL | 102.70-534.90 nmol/L
Evening cortisol	7.50 µg/dL | 206.60 nmol/L	2.90-17.30 µg/dL | 79.90-476.50 nmol/L

## Treatment

The patient was preoperatively prepared with intravenous (IV) hydrocortisone (85 mg, 100 mg/m²/dose). Since the hormonal laboratory workup was negative, no alpha or beta blockers were administered.

**Figure 3.**
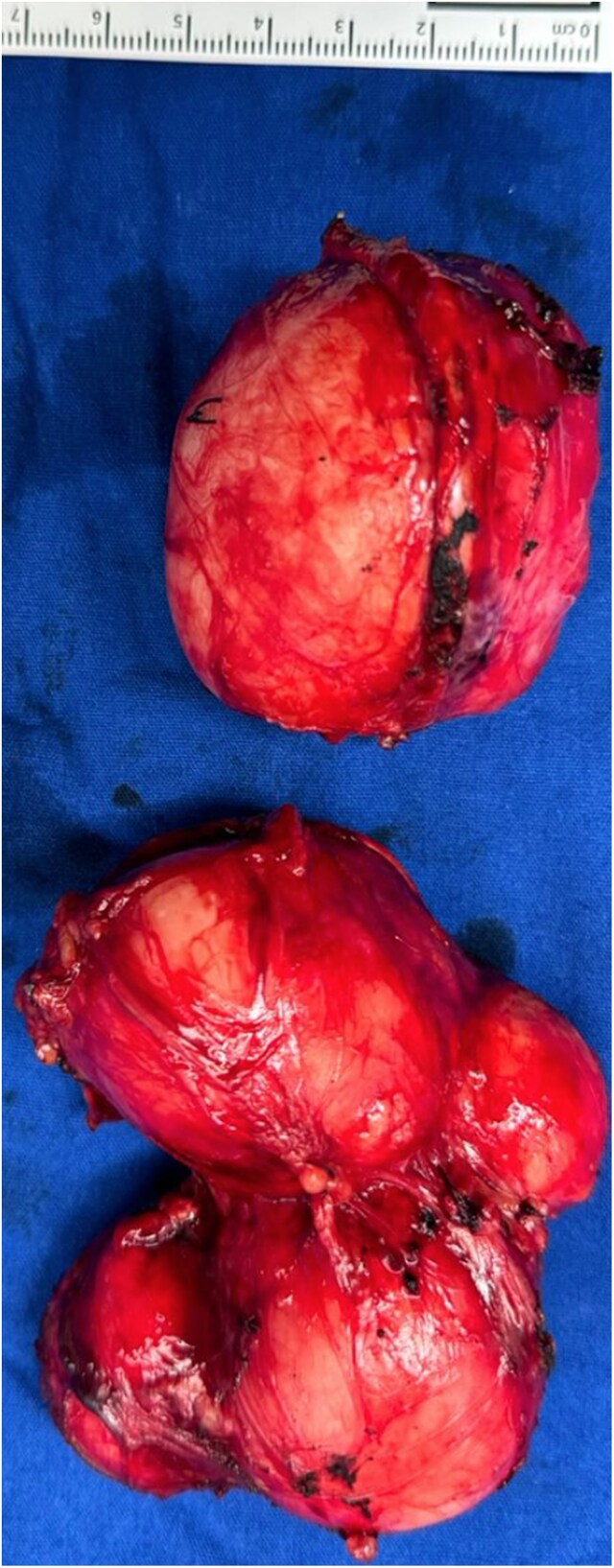
Bilateral adrenal masses: the right adrenal mass measures approximately 6 × 6 cm, while the left adrenal gland contains multiple masses, resulting in distortion of its normal anatomy (approximately 7 × 11 cm in size).

Laparoscopic adrenalectomy was initiated, but due to poor visualization of vascular structures and adherent tissue, a Chevron incision was performed. Retroperitoneal access allowed complete resection of both adrenal tumors. The left tumor measured 11 cm, and the right tumor measured 6 cm [Fig. 3]. Bilateral resection of the masses was performed for 3 reasons: (i) the patient reported abdominal pain, which was thought to be secondary to the masses; (ii) frozen section studies were not available at the hospital; and (iii) to minimize the risk of reintervention.

Postoperative care included IV hydrocortisone (85 mg) in recovery, followed by a maintenance regimen of 100 mg/m²/day, divided into 4 doses given every 6 hours. The dosage was gradually tapered by 20 mg IV every 48 hours. At discharge, the patient was transitioned to oral prednisone at 25 mg/m²/day, divided into 4 doses given every 6 hours, along with 100 micrograms of fludrocortisone daily, and the patient was scheduled for monthly follow-up visits.

## Outcome and Follow-Up

**Figure 4.**
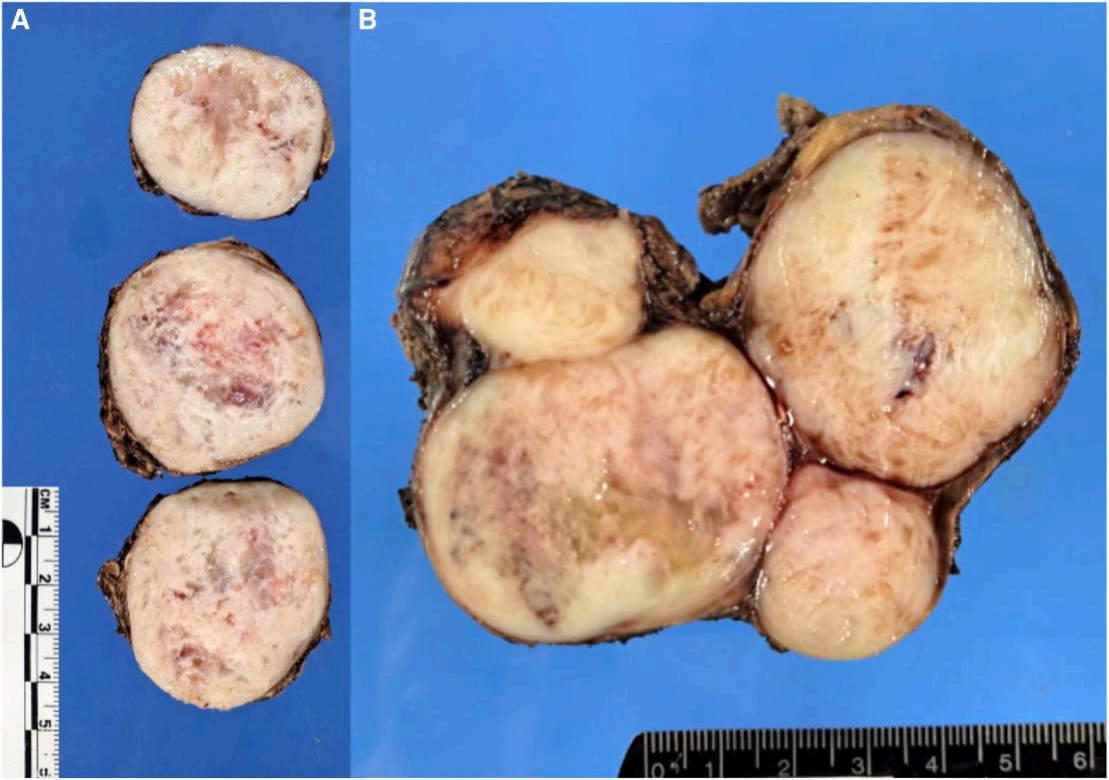
(A): Right adrenal gland with a leiomyoma at its center. (B): Left adrenal gland with leiomyoma at its center.

**Figure 5.**
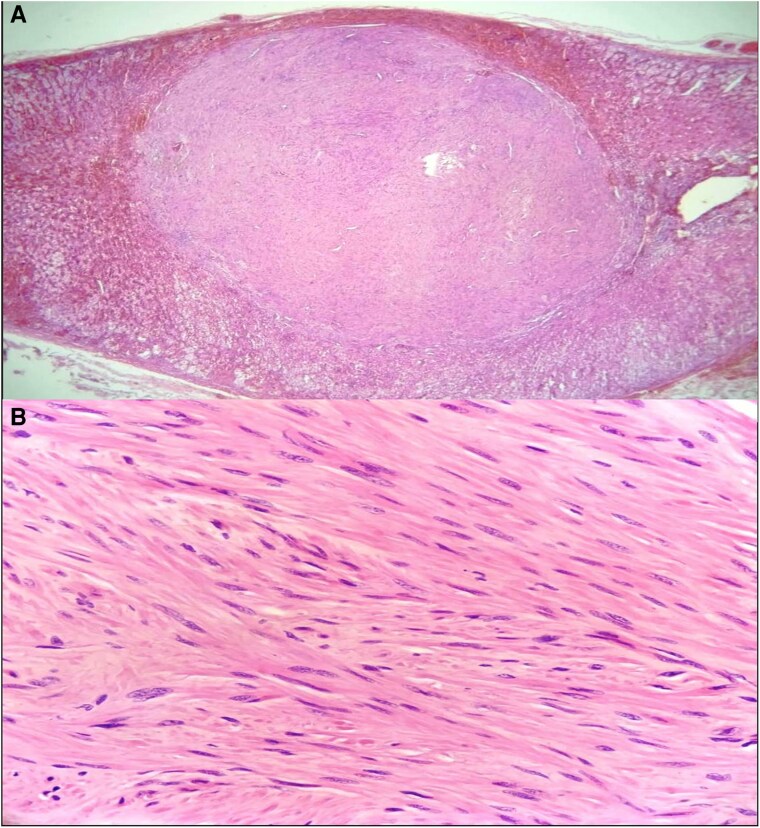
At higher magnification, spindle-shaped cells with elongated, blunt-ended nuclei are observed forming intersecting bundles, consistent with a diagnosis of bilateral adrenal leiomyomas.

Histopathological analysis ruled out the initial clinical impression of carcinoma. Macroscopic examination revealed adrenal gland parenchyma with a leiomyoma at its center [Fig. 4]. Higher magnification revealed spindle-shaped cells with elongated, blunt-ended nuclei forming intersecting bundles, confirming the presence of bilateral adrenal leiomyomas [Fig. 5]. Latent membrane protein 1 (LMP1), an EBV-encoded protein used as a marker in immunohistochemical staining, was negative, making an infection-associated malignancy less likely.

Postoperative recovery was uneventful, and the patient has been doing well 3 months after surgery. He continues to show improvement under corticosteroid replacement therapy. The patient is monitored closely during regular follow-up assessments for signs of complications or recurrence, with ongoing clinical stability.

## Discussion

Adrenal leiomyomas are rare tumors that originate from the smooth muscle cells of the adrenal veins and their tributaries. They are predominantly unilateral, with only 28 cases reported in the literature, 6 of which involved bilateral tumors. Of these bilateral cases, 4 were observed in pediatric patients. Clinical associations for bilateral adrenal leiomyomas include conditions such as multiple vascular leiomyomas, acquired immunodeficiency syndrome (AIDS), large B-cell lymphoma, pulmonary tuberculosis, EBV infection, organ transplantation, or prior chemotherapy exposure [3-5].

The pathogenesis of these tumors is thought to involve an altered immune response. Most adrenal leiomyomas are asymptomatic and metabolically inactive, though partial metabolic activity has been observed in some cases [3-5]. In immunodeficiency states, multifocal involvement and multiple organ lesions are more common. These tumors are often detected incidentally, earning them the classification of incidentalomas, as exemplified by this patient [5, 6].

Imaging plays a pivotal role in diagnosing adrenal leiomyomas. They are typically described as well-circumscribed, round or lobulated masses with poor internal enhancement. Larger tumors may exhibit T2-weighted imaging hyperintensity, internal necrosis, and calcifications. In the present case, calcifications were observed, consistent with the imaging characteristics typically seen. Although fludeoxyglucose (18F)-positron emission tomography (FDG-PET) scans are generally negative for these tumors, increased vascularity can sometimes lead to false-positive PET findings [2-6].

In the fifth edition of the World Health Organization (WHO) classification, EBV-associated leiomyomas are classified as a viral induced smooth muscle tumor. These tumors are usually seen in the context of immunosuppression, post–organ transplantation, or congenital immunodeficiency [7]. This highlights the need for thorough immunological evaluation in patients with bilateral adrenal leiomyomas. However, in this case, the patient tested negative for LMP1, a key marker used to detect EBV in tissues and used to evaluate EBV-associated malignancies. Although this makes an EBV-associated leiomyoma less likely, it does not entirely rule out the pathogen's involvement, as certain cases of EBV-related malignancies may not express this marker, particularly in early stages or in tumors with a muted immune response [7].

Surgical intervention remains the cornerstone of treatment, with laparoscopic adrenalectomy considered the gold standard. This approach is associated with reduced postoperative discomfort, shorter hospital stays, and lower complication rates and is deemed curative in most cases [8]. While some authors recommend limiting laparoscopic adrenalectomy to tumors smaller than 6 cm, successful procedures have been reported for tumors as large as 13 cm [9]. Alternative approaches, such as hand-assisted laparoscopic or open adrenalectomy, may be employed in cases where tumor size, vascular involvement, or other factors complicate visualization during minimally invasive surgery [8-10]. In this case, an open approach (Chevron incision) was required due to difficulties encountered during the laparoscopic procedure.

A limitation in the evaluation of this case was the decision by genetic specialists to forgo genetic testing, despite the patient's elevated IgE levels. Given the clinical context, they concluded that genetic studies were not warranted, which might limit further insight into potential underlying genetic factors. One such factor that could not be fully ruled out is Capping Protein Regulator and Myosin 1 Linker 2 (CARMIL2) deficiency, a condition in which patients typically present with high IgE levels, elevated eosinophils, severe eczema, warts (human papilloma virus and molluscum), Herpesviridae infections, eosinophilic gastrointestinal disease, inflammatory bowel disease, asthma/bronchiectasis, and EBV-associated malignancies. The inability to perform genetic testing leaves the possibility of this rare condition unconfirmed, which may have implications for the patient's broader clinical picture [11].

This case contributes to the limited literature on bilateral adrenal leiomyomas, particularly in pediatric patients. It reinforces the importance of comprehensive diagnostic imaging, immunological workup, and tailored surgical management to achieve favorable outcomes.

## Learning Points

- Adrenal leiomyomas are rare tumors that originate from the smooth muscle cells in the adrenal glands. They are typically benign and occur infrequently in the pediatric population.
- These tumors often present with nonspecific symptoms, such as abdominal pain, weight loss, or malaise. While most cases are nonfunctional, some can present with elevated catecholamine levels, leading to hypertension.
- Imaging plays a crucial role in diagnosing adrenal leiomyomas. Bilateral adrenal masses can be detected on imaging studies, and further diagnostic tests (such as a triphasic CT scan) can help differentiate these tumors from other adrenal conditions. A thorough evaluation of imaging findings is essential for accurate diagnosis.
- Surgical treatment is the gold standard. Laparoscopic adrenalectomy is the preferred method for treating adrenal tumors, offering benefits such as less postoperative discomfort, shorter hospital stays, and a lower rate of complications. It is considered curative, even for tumors of significant size.

## Data Availability

Data sharing is not applicable to this article as no datasets were generated or analyzed during the current study.

## Abbreviations

CT: computed tomography
EBV: Epstein-Barr virus
IV: intravenous
LMP1: latent membrane protein 1
PET: positron emission tomography
